# Human papillomavirus correlates of high grade cervical dysplasia among HIV-Infected women at a major treatment centre in Nigeria: a cross-sectional study

**DOI:** 10.11604/pamj.2019.33.125.17589

**Published:** 2019-06-18

**Authors:** Martin Maimako Yakub, Adeola Fowotade, Chinenye Gloria Anaedobe, Mohammed Mohammed Manga, Rasheed Ajani Bakare, Bawa Ahmed Abimiku

**Affiliations:** 1 Department of Medical Microbiology and Parasitology, University College Hospital, Ibadan, Nigeria; 2 Department of Medical Microbiology, Federal Medical Centre, Nassarawa, Keffi, Nigeria; 3 Department of Medical Microbiology and Parasitology, College of Medicine, University of Ibadan, Ibadan, Nigeria; 4 Department of Medical Microbiology, Gwagwalada Teaching Hospital, Abuja, Nigeria; 5 Department of Medical Microbiology and Immunology, Federal Teaching Hospital Gombe, Gombe State, Nigeria; 6 Department of Histopathology and Forensic Medicine, Gwagwalada Teaching Hospital, Abuja, Nigeria

**Keywords:** Human papillomavirus, genotypes, uterine cervix, premalignant lesions, HIV-positive women, Nigeria

## Abstract

**Introduction:**

Persistent high-risk HPV (hrHPV) infection is higher among women living with HIV/AIDS thus increasing their risk for cervical cancer. We evaluated the virological and immunological correlates of cervical dysplasia in HIV-infected women.

**Methods:**

A cohort of 220 consenting women attending the antiretroviral clinic of the Federal Medical Centre, Keffi, Nigeria was tested for cervical human papilloma virus (HPV) infection using PCR. The prevalent HPV genotypes were determined by DNA sequencing. CD4+T count and type specific HPV was correlated with cervical cytology. Descriptive and inferential statistical analysis of the data was done using the statistical package for social sciences (SPSS) version 20 (SPSS Inc, Illinois, USA) for analysis after validation

**Results:**

Overall HPV prevalence was 54.1% while the hrHPV prevalence was 35.9%. Premalignant and malignant lesions were observed among participants with CD4+T counts between 200-300/mm^3^. A statistically significant association was observed between cervical premalignant lesions and CD4+ count (X^2^=24.747, P value=0.001) as well as hrHPV infections (X^2^=46.800, P<0.001).

**Conclusion:**

Risk stratification with HPV screening among HIV-infected women will help in early case management of cervical precancerous lesions.

## Introduction

Human papilloma virus (HPV) is the most common sexually transmitted pathogen in humans and has been implicated in the aetiology of cervical, oropharyngeal and other anogenital cancers [1,2]. More than 100 HPV genotypes have been characterized and classified based on their oncogenic potential into high-risk HPV (hrHPV) and low-risk HPV (lrHPV) genotypes [1,3,4]. Persistent high risk HPV infection is necessary but requires additional co-factors to cause both malignant and premalignant cervical lesions [1]. The immunosuppressive state induced by Human Immunodeficiency Virus (HIV) potentially increases HPV acquisition, persistence, and development of cervical cancer [2]. Globally, an increased risk of HPV infection and cervical squamous intraepithelial lesions (SIL) has been reported among HIV-positive women [2, 5-7]. Previous studies among HIV-positive women have reported increased multiple infections with varied hrHPV genotypes [1,2,8]. Although cancer of the uterine cervix is the commonest genital tract malignancy in Northern Nigeria, only a few studies have reported the prevalence of type-specific HPV among HIV-infected women [9,10]. We have previously reported a high burden of HPV 1^6^ and 18 and their association with malignant/premalignant cervical lesions among non HIV-infected women in Northern Nigeria. In the present study, we determined the prevalence of hrHPV and levels of CD4+T counts among HIV-positive women attending the Federal Medical Centre (FMC) Keffi and also evaluated the association between both factors and presence of cervical premalignant lesions.

## Methods

### Study area and population

The study was carried out at the antiretroviral clinic of Federal Medical Centre, Keffi in Nigeria between August 2016 and May 201^7^. Eligible women were briefed about the study and were offered on-site cervical cancer screening with conventional Pap smear. HIV positive women were enrolled, if they were aged between 20 and 50 years of age, not pregnant and did not have a history of hysterectomy or cervical cancer. During the enrolment visit, socio-demographic data were collected and cervical sampling for HPV testing was done. CD4+T cell counts and HIV viral load done within six months prior to the study was retrieved from the patients' records. Written informed consent was obtained from all participants. Ethical approval for the study was given by the Research and Ethics Committee of FMC, Keffi (Ref number: Ref: KNH-ERC/01/3618).

### Specimen collection and storage

Rovers^®^ Cervex-Brush^®^ cell sampling device (Rovers Medical Devices B.V 5347 KV Oss, The Netherlands) and liquid based cytological processing and preservative reagent (Zeni-Prep^®^, Zenith diagnostics, USA) were used for specimen collection and transport. Specimen was obtained by inserting the cytobrush into the cervical canal and rotating it four times to collect all the cervical epithelial cells which adhered to the flat sides of the bristles. The brush was then transferred into the vial containing preservative fluid.

### Papanicolaou smear

Cervical smears obtained from each participant was stained using the standard papanicolaou's method in order to assess the presence of cervical premalignant/malignant lesions which was rated according to the 2001 Bethesda system of classification by a cytopathologist.

### DNA extraction

HPV testing was done at the DNA Labs, Kaduna, Nigeria. Gel extraction prior to DNA purification for sequencing was done using QIAquick Gel Extraction Kit (QIAGEN Sample & Assay Technologies, Germany). Briefly, HPV DNA was extracted from exfoliated cervical cells using proteinase K-based digestion protocol. Cells were incubated with proteinase K solution (100 μg/ml) for three hours at 55°C. DNA was then further purified by spin column chromatography.

### Detection and typing of HPV

The extracted DNA was amplified for HPV using a nested PCR approach with GP5+/GP6+( [GP5 + 5¹-TTT GTT ACT GTG GTA GAT ACTAC-3¹], GP6+[5¹- GAAAAATAAACTGTAAATCATATTC-3¹] ) and PGMY 09/11 primer sets (MY/GP(+) [11] that amplify a conserved 150 bp sequence of the L1 open reading frame. AccuPowerHotStart PCR Premix (Bioneer Corporation, South Korea) was used for the PCR while genotypic identification was achieved by direct sequencing using the Gp6+ oligoprimer. Assays were normalized to a reference gene and a calibrator was included in every run. Thermal cycler (BioRad) and Beckman Coultier CEQ 2000XL were used for PCR and sequencing respectively. Sequence alignments were performed against various standard HPV genotype sequences stored in the GenBank database by on-line BLAST analysis to arrive at specific genotyping. HIV diagnosis was confirmed in all participants using the standard national testing algorithm of rapid immunoassays: Uni-Gold™ Recombigen^®^ HIV (Trinity Biotech plc, Bray, Ireland) and Determine^®^ HIV-1/2 (Abbott Japan co Ltd, MinatoKu, Tokyo, Japan). Indeterminate results were subjected to enzyme-linked immunosorbent assay to confirm HIV status.

### Data analysis

Descriptive and inferential statistical analysis of the data was done using the statistical package for social sciences (SPSS) version 20 (SPSS Inc, Illinois, USA) for analysis after validation. Categorical variables were summarized as proportions and further analyzed using Chi square test to assess significance of association between the variables. Logistic regression analysis was done between CD4+T count and cervical premalignant lesions and relationship expressed in odd ratio (OR).

## Results

### Socio-demographic characteristics

A total of 220 HIV-positive women were enrolled. Majority of the participants, 79 (35.9%) were in the age group 30-40 years followed by the age groups; <30 years; 56 (25.5%) and 41-50 years 47 (21.7%) while only 2 (0.9%) were within the age group 61-65 years. Most of the participants, 133 (60.5%) were married while others, 87 (39.5%) were either single or divorced. Most of the participants were Christians, 149 (69.9%) and predominantly from monogamous family setting, 138 (65.4%). Many of the participants, 69 (31.4%) had only primary education, while 67 (30.5%) had no formal education. More than half of the participants, 115 (52.3%) were unemployed and were housewives, while 66 (30%) were self-employed and the remaining 33 (15%) were employed either in the public or private sector. Majority of the participants 215 (97.7%) were already on HAART while the others were treatment naive.

### Analysis of HPV Infection

Overall, HPV DNA was detected in 119 (54.1%) of the participants while high-risk HPV types were detected in 79 (35.9%) samples. HPV 35 was the most frequent 12 (20.5%), with multiple HPV infections seen in 25 (21.0%) samples. Other observed genotypes are listed in Table 1.

**Table 1:** Frequency distribution of HPV genotypes among the participants

HPV genotypes	No of participants	Percentage (%)
HPV6	9	7.1
HPV11	6	4.7
HPV16**	18	14.2
HPV18**	6	4.7
HPV31**	2	1.6
HPV33**	12	9.4
HPV35**	26	20.5
HPV39**	1	0.8
HPV40	1	0.8
HPV42	4	3.1
HPV43	4	3.1
HPV44	1	0.8
HPV45**	17	13.4
HPV51**	1	0.8
HPV52**	1	0.8
HPV56**	4	3.1
HPV66	3	2.4
HPV72	6	4.7
HPV81	5	3.9
Total	119	100.0

Multiple infections were found in 25 (21.0%)

**Key**:

** High risk HPV

### Association between CD4+ count and hrHPV infection

CD4+T counts estimated within six months of sample collection were available for 193 (87.7%) of the participants. Although majority, 91 (47.2%) had CD4+ T values greater than 500 cells/mm^3^, there was no significant statistical association between CD4+T counts and hrHPV infection.

### Cytology findings and hrHPV infection

Follow-up of women with abnormal cytology was obtained in 214 (97.3%) cases with 54 (25.3%) showing premalignant/malignant cytology. The observed premalignant/malignant variants were as follows; invasive squamous cell carcinoma, 1 (0.47%), adenocarcinoma and adenocarcinoma in situ, 1 (0.47%). The others were CIN III, CIN II and CIN I which constituted 6 (2.8%), 23 (10.7%) and 18 (8.4%) respectively. There was a statistically significant association between hrHPV and cervical precancerous lesion (X^2^=46.800, P<0.001**) (Table 2).

**Table 2:** Association between CD4+ count and cervical cytology

	Cervical Cytology				
	Unsatisfactory	Inflammation/ HPV Changes	Premalignant & Malignant Lesion	χ^2^ value	P-value
CD4+ count (cells/mm^3^) <200	Frequency (%)	Frequency (%)	Frequency (%)	27.439	0.000^**^
	2 (12.5)	11 (68.8)	3 (18.8)		
200-300	1 (4.5)	5 (22.7)	16 (72.2)		
301-400	3 (7.7)	23 (59.0)	13 (33.3)		
401-500	2 (7.7)	17 (65.4)	7 (26.9)		
Above 500	11 (12.2)	65 (72.2)	14 (15.6)		
Total	19	121	53		

### Association between CD4+ count and cervical cytology

Premalignant and malignant lesions were observed among participants with CD4+T counts between 200-300 cells/mm^3^. Of the 16 cytology slides reported for participants with CD4+T count less than 200 cells/mm^3^, 3 (18.3%) were premalignant compared with the 22 slides reported for participants with CD4+T count between 200-300 cells/mm^3^, among which 16 (72.2%) were premalignant. Of the 90 slides from participants with CD4+T count greater than 500 cells/mm^3^, only 14 (15.6%) were premalignant. A significant statistical association was found between CD4+T count and cervical premalignant lesions in this study (X^2^=24.747, P value=0.001). The logistic regression model predicted correctly 68.4% of all cases of cervical cytology and also suggested that the CD4+T count is responsible for the 16.5% variation in cervical cytology. Participants whose CD4+T count were less than 200 cell/mm^3^ or ranged between 200 to 300 cell/mm^3^ or between 301 to 400 cells/mm^3^ were more likely to have premalignant lesions than HPV/inflammatory changes {(ODD ratio (OR)=1.266, significance=0.74), (OR=14.857, significance=0.00) and (OR=2.624, significance=0.034) respectively}. This finding implies that these individuals are 1.266 times, 14.657 times and 2,624 times more likely to have premalignant lesions rather than HPV/inflammatory changes in their respective CD4+ T count groups (Table 3).

**Table 3:** Logistic regression of CD4+ count and cervical cytology

Grouped cervical cytology		B	Std. Error	Wald	Df	Sig.	Odd ratio (OR)	95% Confidence Interval for Exp(B)	
								Lower Bound	Upper Bound
**Unsatisfactory/HPV changes**	Intercept	-1.776	.326	29.691	1	.000			
	[CD4-1]	.072	.835	.007	1	.932	1.074	.209	5.520
	[CD4-2]	.167	1.143	.021	1	.884	1.182	.126	11.102
	[CD4-3]	-.260	.695	.140	1	.708	.771	.197	3.010
	[CD4-4]	-.364	.816	.199	1	.656	.695	.141	3.438
	[CD4-5]	0	.	.	0	.	.	.	.
**Premalignant & malignant lesion**	Intercept	-1.535	.295	27.153	1	.000			
	[CD4-1]	.236	.715	.109	1	.741	1.266	.312	5.141
	[CD4-2]	2.698	.591	20.846	1	.000	14.857	4.665	47.317
	[CD4-3]	.965	.455	4.492	1	.034	2.624	1.075	6.404
	[CD4-4]	.648	.537	1.456	1	.228	1.912	.667	5.478
	[CD4-5]	0	.	.	0	.	.	.	.

Key

CD4-1= < 200 cell/mm^3^

CD4-2 = 200-300 cell/mm^3^

CD4-3 =301-400 cells/mm^3^

CD4-4 = 401-500 cell/mm^3^

CD4-5 >500 cell/mm^3^

## Discussion

This study reveals a high cervical HPV prevalence of 54.1% among HIV positive women attending the antiretroviral clinic in Keffi, North-central Nigeria. This finding is higher than the 48.1% prevalence of cervical HPV infection observed in our previous study among women from Gombe, North-eastern Nigeria [12]. This is however not surprising as the previous study was conducted among HIV-negative women who presented for routine cervical cancer screening. The high prevalence of hrHPV (35.9%) among the studied HIV-infected women conforms to the 36% reported by Akarolo-Anthony *et al*. in a study conducted in Abuja to determine HIV-associated hrHPV infection among Nigerian women [13]. Higher prevalence rates of hrHPV has previously been reported from other parts of Africa, including the 52.6% reported in a similar study conducted by De Vuyst *et al*. among HIV positive women in Kenya [14] and the 52.4% reported by McDonald *et al*. among HIV-positive and HIV-negative women in Cape Town, South Africa [15]. This disparity may be explained by the higher national prevalence of HIV in Kenya [14]. Epidemiological reports from developed countries have also revealed higher hrHPV prevalence rates such as the 43% reported by Konopricki *et al*. in a study conducted to determine hrHPV infection among HIV positive women living in Europe [16]. The observed differences in the prevalence of HPV infection may be attributed to the geographical variations as well as differences in socio-economic class, level of education and individual sexual risk behaviours.

The five most predominant hrHPV genotypes were; HPV 35, 16, 45, 33, and 18. This result is consistent with findings from previous similar studies in Nigeria, which reported findings of non-16 and non-18 genotypes among HIV positive women [13,17]. A previous study from Abuja, North-central, Nigeria identified predominantly genotypes 35, 56, 58, 59 and 45 [12] while genotypes 31, 52, 53 and 35 were the predominant genotypes from a similar study in Lagos, South-west, Nigeria [17]. Variation in lifestyle, socio-cultural characteristics, environment, sexual behaviour and genetic factors across various parts of Nigeria might account for the observed disparity in prevalent HPV genotypes. Notably, the predominant HPV types identified from our study are among the five predominant HPV types (16, 18, 31, 33 and 45) associated with 80% of cervical cancers [18]. Multiple HPV infection observed in 22.5% of the participants might be due to persistent HPV infections from multiple sexual partners with poor virus clearance as a result of HIV immunosuppression. The multiple infection rates are in conformity with similar studies among HIV-infected women from Nigeria and other parts of the world including Kenya [14], Italy [19] and the USA [20].

In this study, 68.4% of hrHPV was associated with different grades of premalignant/malignant lesions while none of the low risk HPV was associated with premalignant lesions hence the significant statistical association found between hrHPV infection and precancerous lesions. A similar study by our group among non-HIV infected women in Gombe, North-east Nigeria did not show significant association between HPV infection and cervical cytological changes [12]. This disparity might be due to variation in the sexual risk factors as well as the HIV status of the participants.

The cytology report of 25% of the participants showed precancerous/cancerous cervical lesions, of which 5 (9.2%) were invasive squamous cell carcinomas and 1 (1.9%) was adenocarcinoma type of cervical cancer. This finding is consistent with reports from other sub-Saharan African countries where varied rates of 33%, 26.7%, 24.3%, 66.3% and 73.0% were reported from Zambia, Kenya, Rwanda, South Africa and Uganda respectively [21-25]. However, lower prevalence rates of 16% and 19% had previously been reported from Mumbai, India [26] and Yunnan Province of China [27] respectively. These figures suggest that the high burden of cervical cancer in most African countries might be due to the high prevalence of HIV in sub-Saharan Africa. Risk stratification with HPV screening will help with early case detection and treatment for cervical precancerous lesions among HIV-infected women.

In the present study, the risk of cervical precancerous lesions was higher in patients with lower CD4+T counts and there was a statistical significant association between CD4+T and cervical precancerous lesion. Participants whose CD4+T counts ranged between 200-300 cells/mm^3^ had more premalignant lesions and the risk of developing premalignant lesions decreased with increasing CD4+T counts. This finding conforms to similar reports from Kenya and China, where a significant association was observed between CD4+T count and cervical precancerous lesions [22,27]. The high percentage of cervical inflammatory lesions observed among the studied women suggests possible co-infection with other Sexually transmitted diseases (STDs) as co-infection with other STDs is a known significant facilitating factor for HPV infection [28,29].

## Conclusion

The current study highlights high hrHPV prevalence of 35.9% and among HIV-infected women. Nineteen different HPV types were detected with the five most predominant types (sequentially) being 16, 18, 31, 33 and 45. Our finding suggests that high risk HPV infection is an independent risk factor for cervical precancerous lesion. Additionally, HIV positive women with low CD4+T count are at a higher risk of cervical precancerous lesions. The high prevalence of precancerous lesions in the HIV-infected sub-population justifies the need for routine targeted screening and treatment of HIV-infected women to reduce morbidity and mortality from cervical cancer.

### What is known about this topic

- Multiple studies have shown that HIV-positive women are significantly at a higher risk of developing cervical cancer;
- The incidence of cervical cancer in women with HIV compared with women without HIV is highest in low and middle-income countries;
- Majority of invasive cervical cancer are preventable with improved access to high-functioning HPV testing and other cervical cancer screening modalities would substantially decrease the burden of cervical cancer for women living with and without HIV.

### What this study adds

- Levels of CD4+ count and hrHPV infection are important determinants of cervical premalignant lesions;
- The most prevalent HPV genotype (HPV 35), is not covered by the currently available quadrivalent and nonavalent HPV vaccines;
- The study therefore justified the need for routine targeted screening and treatment of HIV-infected women to reduce morbidity and mortality from cervical cancer.

## Competing interests

The authors declare no competing interests.
